# Synergistic Impairment of the Neurovascular Unit by HIV-1 Infection and Methamphetamine Use: Implications for HIV-1-Associated Neurocognitive Disorders

**DOI:** 10.3390/v13091883

**Published:** 2021-09-21

**Authors:** Nikolai Fattakhov, Silvia Torices, Michael Stangis, Minseon Park, Michal Toborek

**Affiliations:** 1 Department of Biochemistry and Molecular Biology, Miller School of Medicine, University of Miami, Miami, FL 33136, USA; sxt736@med.miami.edu (S.T.); mstangis@miami.edu (M.S.); mspark@med.miami.edu (M.P.); 2 Institute of Physiotherapy and Health Sciences, The Jerzy Kukuczka Academy of Physical Education, 40065 Katowice, Poland

**Keywords:** HIV, methamphetamine, neurovascular unit, HIV-associated neurocognitive disorder, blood–brain barrier, neuroinflammation

## Abstract

The neurovascular units (NVU) are the minimal functional units of the blood–brain barrier (BBB), composed of endothelial cells, pericytes, astrocytes, microglia, neurons, and the basement membrane. The BBB serves as an important interface for immune communication between the brain and peripheral circulation. Disruption of the NVU by the human immunodeficiency virus-1 (HIV-1) induces dysfunction of the BBB and triggers inflammatory responses, which can lead to the development of neurocognitive impairments collectively known as HIV-1-associated neurocognitive disorders (HAND). Methamphetamine (METH) use disorder is a frequent comorbidity among individuals infected with HIV-1. METH use may be associated not only with rapid HIV-1 disease progression but also with accelerated onset and increased severity of HAND. However, the molecular mechanisms of METH-induced neuronal injury and cognitive impairment in the context of HIV-1 infection are poorly understood. In this review, we summarize recent progress in the signaling pathways mediating synergistic impairment of the BBB and neuronal injury induced by METH and HIV-1, potentially accelerating the onset or severity of HAND in HIV-1-positive METH abusers. We also discuss potential therapies to limit neuroinflammation and NVU damage in HIV-1-infected METH abusers.

## 1. Introduction

At the end of 2019, there were an estimated 38 million people living with human immunodeficiency virus (HIV) globally [1]. The introduction of antiretroviral therapy (ART) has significantly transformed the face of HIV type 1 (HIV-1) infection, from a terminal illness to a chronic manageable disease. However, HIV-1-associated neurocognitive disorder (HAND), which ranges from asymptomatic neurocognitive impairments to severe dementia, remains highly prevalent in HIV-1-infected populations, affecting activities of daily living [2]. HAND develops through the initial infiltration of HIV-1-infected immune cells into the brain by crossing the blood–brain barrier (BBB), followed by the initiation of pathogenetic processes, including neuroinflammation, and the establishment of compartmentalized HIV-1 replication in the cells of the neurovascular unit (NVU) including microglia, astrocytes, and pericytes [3,4,5,6]. Although endothelial cells and neurons are not permissive to active HIV-1 infection and replication [7], their dysfunction is likely to be caused by indirect pathogenic mechanisms such as HIV-1-altered oxidative balance, leading to increased reactive oxygen species (ROS) generation [8,9]. In addition, HIV-1 proteins, including glycoprotein (gp) 120, transactivator of transcription (Tat), and negative regulator factor (Nef), along with elevated levels of proinflammatory mediators can either directly or indirectly lead to neuronal cell death contributing to the persistence of HAND [10,11,12].

**Figure 1:**
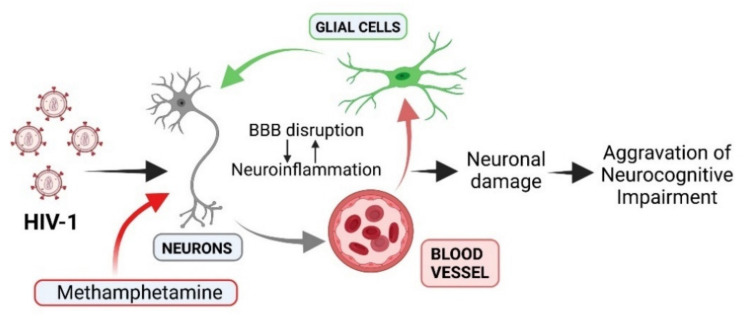
A schematic diagram illustrating a possible contribution of neurovascular unit (NVU) disruption to increased neurocognitive impairment in comorbid HIV-1 infection and methamphetamine (METH) use. The NVU is composed of vascular cells, glial cells, and neurons. METH use and HIV-1 infection compromise blood–brain barrier (BBB) integrity facilitating transmigration of infected immune cells and viral entry across the BBB. This can potentially lead to neuroinflammatory responses by microglia that can further promote BBB disruption. These events result in worsening neuronal damage and aggravating neurocognitive impairments involved in the pathogenesis of HIV-1-associated neurocognitive disorders (HAND). Created with BioRender.com.

Methamphetamine (METH) is a highly addictive psychostimulant drug, the abuse of which has reached epidemic proportions worldwide, with its use being particularly high among people living with HIV-1 [13]. METH abuse raises the risk of contracting or transmitting HIV-1 and other blood-borne viruses, not only for individuals who inject the drug, but also for noninjecting METH users, as it is associated with an increase in risky sexual behavior [14,15,16,17]. METH use, either alone or in combination with other drugs, is associated with failure of viral suppression among HIV-1-seropositive persons on ART, independent of adherence and sociodemographic factors [18,19]. Recent findings indicate that HIV-1-infected individuals who engage in METH use display exacerbations in key pathophysiologic processes that are linked to faster clinical HIV-1 progression [20]. A growing body of evidence suggests that the combination of HIV-1 infection and METH dependence causes more profound neurocognitive deficits and structural brain abnormalities than either condition alone [21,22]. Results of a recent study deciphering epigenetic signatures of METH dependence in the human prefrontal cortex of HIV-1-positive individuals suggest that METH use increases genomic activities to become transcriptional events related to neurodegeneration, dopaminergic deficits, and decreased cognitive and executive functions [23]. Considering that BBB breakdown can serve as an early biomarker of human cognitive dysfunction, independent of neurodegeneration markers [24,25], we seek to review how METH abuse may exacerbate BBB breakdown and neuroinflammation within the NVU, resulting in the augmentation of neuronal injuries and worsening of neurocognitive impairment in HIV-1-infected individuals (Figure 1).

The current review aims to summarize the findings from recent human and animal studies which might provide new insights into the pathogenesis of HAND in the context of METH dependence. We will not exhaustively review the literature regarding the impact of HIV-1 infection or METH alone on the NVU, because several excellent reviews summarizing the effects of these factors have recently been published [26,27,28,29]. Rather, we will focus on the synergistic molecular mechanisms involved in the neurobehavioral implications of concurrent HIV-1 infection and METH use. The roles of microglia activation and astrocyte dysfunction in comorbid condition will also be discussed, and we will cover potential therapeutic strategies to target neurocognitive impairments and minimize NVU dysfunction in HIV-1-infected METH abusers.

## 2. Synergistic Impairment of the NVU Induced by Comorbid HIV-1 Infection and Methamphetamine Use

The NVU incorporates cellular and extracellular components involved in regulating cerebral blood flow and BBB function [30]. The studies showing evidence of synergistic effects of the combination of METH with HIV-1 or HIV-1 neurotoxic proteins on the components of NVU are presented in Table 1. This Table lists in vivo, in vitro, combined in vivo and in vitro studies, as well as postmortem human brain ex vivo studies with indication of viral inoculum dose/route, METH dosing regimen, and experimental model including genetic background of the animals. The chronic METH use pattern including escalating-dose multiple-binge METH regimen was used in all animal studies to more closely mimic a human usage pattern and cause long-term neuronal damage similar to those observed in human METH abusers. Considering that concentrations of HIV-1 proteins used in many in vitro studies are frequently greater than levels found in the cerebrospinal fluid from individuals with HIV-1 infection [31,32], these research findings should be interpreted in the context of the limitations of the experimental paradigms that do not fully mimic pathophysiological changes associated with HIV-1 infection. While some animal studies have utilized simian immunodeficiency virus (SIV) macaque model and mouse model of HIV-1 infection (EcoHIV), other studies rely on models that are solely based on HIV-1 protein toxicity. These models include HIV-1 transgenic (tg) rat and mouse models, intracerebroventricular or intravenous administration of Tat, and tg mouse models expressing a single viral protein, such as Tat or gp120. Non-inducible HIV-1 mice express these viral proteins throughout their lifespan, which can potentially prevent a normal or healthy development before onset of the desired pathological phenotype [33]. The models that are based on a single HIV-1 protein may miss pathological effects that originate from the combined action of different viral proteins encoded by the HIV-1 genome. In general, mouse models that rely on protein toxicity are not infectious and do not reflect the full myriad of HIV-1 infection.

### 2.1. Disruption of Tight Junctions and Transporters of Brain Endothelial Cells

The BBB is a complex structure composed of a tightly sealed monolayer of brain microvascular endothelial cells (BMECs) endowed with barrier properties, the basement membrane, and other NVU cells, including astrocytes and pericytes that wrap the abluminal capillary surface and provide physical support and stability to the BBB [54]. One of the primary properties of the BBB is the strict regulation of paracellular permeability, due to the presence of junctional complexes, which include tight junctions (TJs), adhering junctions, and gap junctions. TJs form the basic structure of the BBB and are composed of three major classes of transmembrane proteins, including occludin, claudins and junctional adhesion molecules, and additional scaffold proteins. Occludin maintains cell surface polarity through interactions with scaffold protein zonula occludens 1 (ZO-1) and contributes to the permeability of endothelial cells [55]. Junctional adhesion molecule A (JAM-A) is an integral transmembrane protein with numerous functions, including restricting vascular permeability by enhancing the expression of claudin-5, the most enriched TJ protein [56]. Alterations in expression and/or localization of any individual constituent TJ proteins, but primarily claudin-5, can significantly affect BBB properties, particularly barrier permeability, which indicates their critical importance for the maintenance of the central nervous system (CNS) homeostasis [57]. For example, decreased expression or redistribution of claudin-5 has been implicated in neurodegenerative and neuroinflammatory disorders, as well as in psychiatric diseases [58,59].

**Figure 2:**
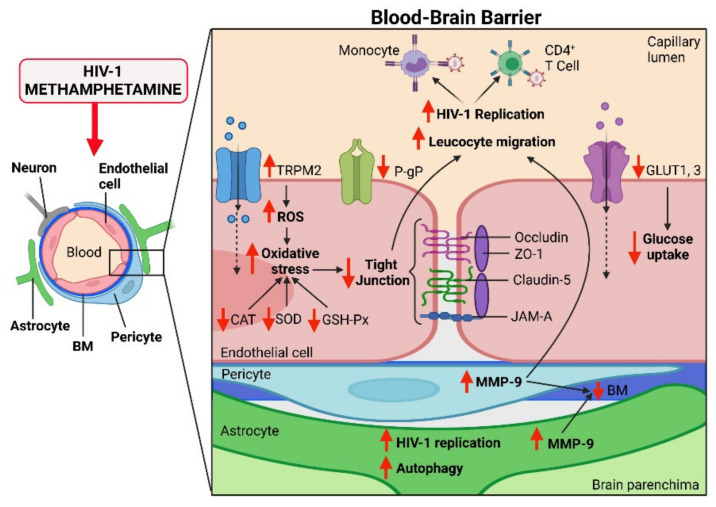
Synergistic impact of HIV-1 infection and methamphetamine (METH) use on the blood–brain barrier (BBB). The main component of the BBB is endothelial cells, which are bridged by tight junction (TJ) protein complexes. Illustrated are four critical TJ proteins that are synergistically affected by comorbid HIV-1 infection and METH: occludin, zonula occludens 1 (ZO-1), claudin-5, and junctional adhesion molecule-A (JAM-A). In addition, HIV-1 infection and METH synergistically affect carrier-mediated transport across the BBB by impairing the functions of P-glycoprotein (P-gp) and two glucose transport proteins, GLUT1 and GLUT3. METH and HIV-1 also synergistically induce oxidative stress via activation of transient receptor potential melastatin 2 (TRPM2) channels in endothelial cells. The combination of METH and HIV-1 infection induces excessive production of reactive oxygen species (ROS) and impairs the defensive abilities of antioxidant enzymes catalase (CAT), glutathione peroxidase (GSH-PX), and superoxide dismutase (SOD). Elevated activity of matrix metalloproteinase-9 (MMP-9) leads to the degradation of the basement membrane (BM). Increased BBB permeability due to degradation of TJs and BM proteins may facilitate transmigration of HIV-1-infected monocytes and CD4+ T cells into the brain. METH enhances HIV-1 replication in HIV-1 infected CD4+ T cells, likely stimulating transmigration of infected immune cells across the BBB. HIV-1 replication can be enhanced by METH, contributing to increased viral load in the brain. Created with BioRender.com.

To enter the CNS, METH and HIV-1 must first break through the BBB. Interestingly, METH and HIV-1 appear to compromise BBB integrity in a synergistic manner (Figure 2). Recent studies revealed that HIV-1 Tat and METH are able to synergistically damage the BBB by decreasing expression of TJ proteins including occludin, claudin-5, ZO-1, and JAM-A in the cerebral cortexes of tree shrews and rats [34,35,39]. Moreover, the combination of HIV-1 Tat and METH further increased permeability of the BBB in vivo, as determined by the accumulation levels of Evans blue and fluorescein sodium in these studies. On the other hand, a study on METH self-administering HIV-tg rats did not reveal any potentiation of BBB damage in the hippocampus [60], a brain region highly vulnerable to HIV-1 infection [61] and METH toxicity [62]. Nevertheless, there were trends for METH to potentiate the impact of Tat on the levels of claudin-5 in that study. Consistent with in vivo findings, exposure of the human brain capillary endothelial cell line HCMEC/D3, representing a well-established in vitro BBB model [63], to the combination of HIV-1 Tat and METH concentrations of 500 μM or higher confirmed the downregulation of occludin, JAM-A, and ZO-1 TJ proteins [34,35]. However, these in vitro studies reporting BBB breakdown employ METH at concentrations that are in excess of the range of METH plasma concentrations in drug abusers. In fact, such high METH levels can be associated with lethality [64]. The exposure of Tat-treated HCMEC/D3 cells to a lower dose of METH (10 μM) that is likely to reflect relevant METH plasma levels resulted only in a decrease in ZO-1 levels, promoting reduced BBB integrity [47]. On the other hand, METH can accumulate in the brain [65,66]; therefore, higher concentrations of METH in studies that employ astrocytes or microglial cells appear to be more appropriate. The mechanisms of HIV-1 and/or METH-mediated disruption of TJ integrity are now fully understood; however, they may involve alterations of Rho signaling [67], enhanced matrix metalloproteinase activity [68], alterations of the Arp2/3 complex-regulated actin rearrangement [69], and induction of oxidative stress [70]. Indeed, it is very well accepted that endothelial cells are sensitive to oxidative stress-induced dysfunction [71,72]. 

The presence of numerous luminal and abluminal protein transporters, solute and ion transporters, and efflux transporters strictly controls the entry and exit of molecules and metabolic by-products across the BBB. It has been shown that HIV-1 Tat protein and METH synergistically impaired the functions of two major glucose transport proteins, GLUT1 and GLUT3 in the brain endothelium of tree shrews [34]. This resulted in decreased glucose uptake and contributed to the disruption of BBB integrity and function. In the brain, GLUT1 predominantly mediates glucose transport across the BBB into astrocytes, whereas GLUT3 provides glucose to neurons [73,74]. Therefore, the impairment of GLUT1 at the brain endothelium caused by METH may contribute to energy-associated disruption of TJ assembly and loss of BBB integrity [75]. Influx and efflux transporters, which extrude toxic metabolites from endothelial cells and limit CNS entry of xenobiotics and therapeutics from the bloodstream, are also a prominent feature of the BBB. Despite the considerable number of studies focused on transport mechanisms that limit BBB drug penetration, it is uncertain the extent to which HIV-1- and METH-induced alterations in ART penetration into the brain resulted from compromised BBB integrity or disrupted efflux transporter function. Several antiretroviral drugs used in the treatment of HIV-1 infection are substrates for P-glycoprotein (P-gp), a major adenosine triphosphate (ATP)-binding cassette transporter, responsible for the extrusion of substrates from the brain back into the blood. One recent study showed that exposure of HCMEC/D3 cells to a combination of Tat and METH resulted in increased cellular accumulation of the P-gp substrate rhodamine 123, suggesting that P-gp was inhibited or that its function was impaired within these cells [47]. Interestingly, the expression and efflux function of P-gp has been reported to be upregulated by Tat [76]. This might explain why HIV-1-infected individuals maintained on suppressive therapy present with low levels of ART in the brain [77]. A better understanding of the mechanism of combined impact of HIV-1 and METH on P-gp may offer solutions to improve the efficiency of ART to reach the brain HIV-1 reservoirs or to enhance the ability of ART to cross the BBB.

### 2.2. Oxidative Stress and Inflammation

Clinical evidence shows that exposure of endothelial cells to HIV-1 is linked to endothelial dysfunction [7]. Molecular in vitro and in vivo studies have shown that this is at least partially mediated by the impact of HIV-1 cellular infection and HIV-1 proteins on the enzymatic sources of oxidative stress in the vascular wall [27,78]. The role of individual effects of METH in the disruption of BBB function by induction of oxidative stress and inflammatory signaling has been also widely recognized [70,79]. A recent study examining how METH can affect the metabolic pathways within the NVU indicated that endothelial cells and neural cells exhibited much higher levels of cellular stress compared to astrocytes and pericytes [80]. A proteome analysis from this study revealed that METH can upregulate stress-activated protein kinase/c-Jun N-terminal kinase (SAPK/JNK) activation pathways, inflammatory pathways, and growth factor signaling pathways including platelet-derived growth factor (PDGF) signaling in human primary BMECs. Results from tree shrew experiments showed that METH and HIV-1 Tat co-induced the oxidative stress response at the BBB, reducing catalase (CAT), glutathione peroxidase (GSH-PX), and superoxide dismutase (SOD) activity, as well as increasing ROS levels [35]. In addition, abnormal endothelial cell morphology and increased apoptosis were found in that study. It was proposed that synergistic elevation of the expression of transient receptor potential melastatin 2 (TRPM2) in the prefrontal cortex contributed to the observed effects [35]. Indeed, TRPM2 channels can mediate the oxidative stress reactions via activation of nucleotide-binding oligomerization domain-like receptor family pyrin domain, containing 3 (NLRP3) inflammasomes, microglia activation, or inducing production of tumor necrosis factor-α [81,82]. Oxidative stress, in turn, can cause a rapid induction of autophagy in human brain vascular pericytes by tyrosine nitration of TRPM2, negatively affecting the BBB [83].

### 2.3. Alterations in Astrocytes and Pericytes

In vivo studies have failed to demonstrate HIV-1 replication in endothelial cells, suggesting that the mechanism accounting for endothelial damage may partially rely on the indirect action of molecules released to the microenvironment by other infected cells in the brain [7], such as astrocytes and pericytes. Astrocyte endfeet cover over 90% of the endothelial cell surface, and release specific factors that modulate the permeability of the BBB [84,85]. Astrocyte loss in the adult mouse brain has been shown to cause BBB damage of varying extents [86,87]. Accumulating evidence suggests that astrocytes support productive HIV-1 infection in vivo [4]. A significant increase in the release of HIV-1 p24 protein by primary human astrocytes was observed upon METH treatment [88], suggesting a role for METH in enhancing viral replication in astrocytes. Another recent study revealed that METH and HIV-1 gp120 caused an increase in levels of the autophagosomal marker LC3-II in astrocytes, and the level of LC3-II was further increased when the cells were treated with METH and gp120 in combination [48]. However, prolonged concurrent treatment exacerbated astrocyte death accompanied by the inhibition of autophagy, suggesting that autophagy might function as a protective response against apoptosis caused by a combined impact of METH and gp120. 

Pericytes are a type of vascular cell that wrap the cerebral capillary walls and play a critical role in the regulation of the BBB [89]. Signals from brain pericytes regulate the expression of genes in endothelial cells that influence transendothelial permeability and enhance astrocyte endfeet/endothelial contacts [90]. BBB pericytes can be productively infected with HIV-1 and are prone to establishing a latent infection in the brain [3]. Dysfunctional pericyte-endothelial interactions and pericyte loss have been suggested to contribute to SIV-induced BBB disruption and neuropathogenesis in HAND [91,92]. Studies from our laboratory also demonstrated that the propagation of HIV-1 infection in human brain pericytes is mediated by enhancing gap junction-mediated intercellular communication and may contribute to BBB dysfunction [93]. Moreover, our recent work suggests that occludin can control HIV-1 transcription in brain pericytes via regulation of sirtuin-1 activation and cooperation with caveolin-1 and Alix [94,95].

The toxic effects of METH on pericytes have been previously reported in several in vitro studies. For example, METH has been shown to increase the migration of pericytes from the endothelial basement membrane via activation of p53 upregulated modulator of apoptosis and sigma-1 receptor [96]. Another study identified that the inflammatory pathways were not activated in human brain pericytes upon METH exposure, while signaling pathways involving nerve growth factor (NGF) and fibroblast growth factors (FGF) were upregulated [80]. We did not identify any published research that linked HIV-1 infection and METH use in brain pericytes. It has recently been discovered that primary brain pericytes infected with HIV-1 are highly susceptible to DNA damage in the presence of glutamate [97]. Based on this information, we hypothesize that increased extracellular glutamate levels seen in HIV-1 and METH comorbidity [49] might contribute to reduced pericyte coverage in this condition.

### 2.4. Facilitation of Immune Cell Transmigration across the BBB

A number of previous studies have shown that METH has the ability to disrupt immune homeostasis, affecting diverse leukocyte subsets, and thereby making METH users more susceptible to HIV-1 infection [98]. In humans, significantly higher viral loads have been reported in the plasma of HIV-1-infected subjects with a history of METH abuse, as compared with HIV-1-infected individuals not using METH [99]. METH has been shown to elevate monocyte activation [100] and enhance HIV-1 replication in monocyte-derived macrophages [101] and CD4^+^ T-cells in vitro [102,103], the primary targets for the virus. Since HIV-1 and METH can independently negatively affect BBB integrity and permeability, combined HIV-1 and METH insults may facilitate leukocyte extravasation from blood to brain. Increased brain viral load was found in rhesus monkeys infected with SIV and exposed to METH [104]. METH has been suggested to increase infectivity of myeloid cells in the brain by stimulating dopamine release and modulating the expression of HIV-1 co-receptor CCR5 [105]. A recent study revealed that HIV-1 proteins and METH independently increase matrix metalloproteinase (MMP)-9 in the NVU, possibly through the activation of nuclear factor-κB (NF-κB) [60]. MMP-9 is a protease involved in the degradation of TJs [106], basement membrane proteins [107], and dystroglycan in the astrocyte endfeet [108]. Cyclooxygenase (COX)-2 might be another common target for the detrimental effects of both METH and HIV-1. This enzyme has been revealed to be implicated in compromising BBB integrity by HIV-1 Tat [109,110] and METH [111]. Nevertheless, more efforts need to be directed toward unveiling the signaling pathways that orchestrate leukocyte–endothelial interactions and how they may converge synergistically during HIV-1 and METH comorbidity.

## 3. Potentiation of Neuroinflammation by the Combination of HIV-1 Infection and Methamphetamine Use

### 3.1. Mechanisms of Neuronal Dysfunction

**Figure 3:**
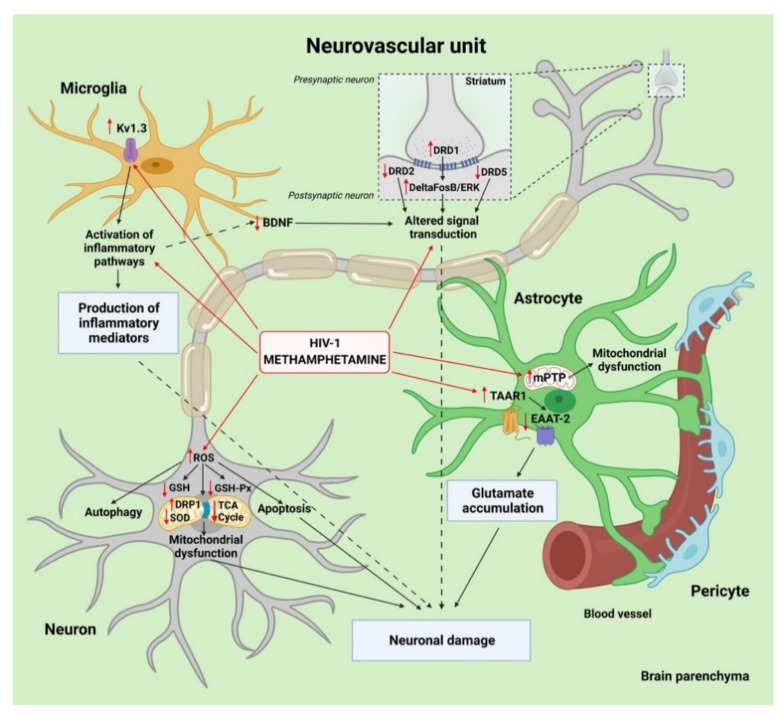
Synergistic cooperation between HIV-1 and methamphetamine (METH) in the neurovascular unit (NVU) potentiates neuroinflammation and enhances neuronal damage. HIV-1 enters the brain via infected monocytes and CD4+ lymphocytes that cross the blood brain barrier (BBB). Once inside the brain, infected macrophages facilitate productive infection and release free virions into the brain parenchyma that infect neighboring microglia and, to some degree, astrocytes and pericytes. This infection leads to the release of neurotoxic factors and to enhanced activation of microglia. METH readily crosses the BBB due to its small size and significantly potentiates neuronal damage, mediated by microglia activation of voltage-gated potassium channel KV1.3. METH also downregulates the release of brain-derived neurotrophic factor (BDNF) from HIV-1-infected microglia. METH and HIV-1 cause astrocyte dysfunction by the opening of mitochondrial permeability transition pores (mPTPs) and modulating TAAR1/EAAT2 signaling pathways involved in glutamate clearance from the extracellular space. The other synergistic effects of METH and HIV-1 include mitochondrial fission mediated by dynamin-related protein 1 (DRP1) and impairment of the tricarboxylic acid (TCA) cycle in neurons. Furthermore, the combination of METH and HIV-1 induces neuronal oxidative damage by downregulating levels of glutathione (GSH), antioxidant defense enzymes glutathione peroxidase (GSH-PX), and superoxide dismutase (SOD). Created with BioRender.com.

While neuronal injury and loss are at the heart of HAND, HIV-1 does not directly injure neurons by productive infection, but rather by indirect or inflammatory factors released from infected cells in the CNS [9]. HIV-1 infection and METH dependence can amplify neuroinflammation by fostering a dysfunctional crosstalk of neurons with microglia and astrocytes (Figure 3). Neurons communicate via unique structures, dendrites, and axons, to receive and send the electrical signals that make neuronal communication possible. Therefore, unraveling the combined effects of HIV-1 and METH on neural connectivity is of great significance. It has been reported that the cortical neurons of rats treated with a combination of Tat and METH exhibited edematous swelling of axonal membranes, which was larger than in the METH and Tat treated groups [39]. This axon injury, in turn, could affect action potential signal transmission [112] or lead to a reduction in nerve conduction velocity [113]. Besides, gp120 and Tat in combination with METH induced focal swellings of dendrites and reduced the total length of the neuritic network in human primary neurons [50]. Data from animal studies suggested that loss or alteration of dendritic spines resulting from METH treatment of HIV-1 gp120 tg mice led to impaired learning and memory [40]. Moreover, there was a marked reduction in staining for microtubule associated protein (MAP)-2, expressed mainly in dendritic extensions in the human primary neurons exposed to HIV-1 expressing microglia cells [114]. In contrast, a large study of postmortem human HIV-1-infected brains from subjects with long-term METH dependence revealed no significant association of METH with dendritic spine loss in the cortex [22].

Recent studies have demonstrated that chronic exposure to METH exacerbates Tat-induced neurotoxicity, partially due to causing excessive ROS production and impairing antioxidant systems [115]. For example, a study based on human SH-SY5Y neuroblastoma cells revealed a strong synergistic effect of the combined METH and Tat treatment on ROS production and the inhibition of protein expression levels of GSH-PX and SOD, key players in antioxidant defense [36]. This study has also provided experimental evidence that concurrent exposure to Tat and METH resulted in greater apoptosis than METH or Tat treatment alone in vivo and in vitro. Another study found that METH can synergistically induce autophagy in primary midbrain neuronal cells of tree shrews [51]. It was also demonstrated that Tat and METH have an additive effect in lowering the levels of glutathione (GSH), which plays critical roles in maintaining the cellular redox status and protecting cells from oxidative damage [39].

Neural tissues depend on mitochondria to produce ATP via the tricarboxylic acid (TCA) cycle, and oxidative phosphorylation (OXPHOS) required for mechanisms that ultimately determine neuronal performance, such as excitability and synaptic signaling [116]. Excessive ROS generation causes oxidative stress injuries not only in the form of lipid peroxidation, protein peroxidation, and DNA damage, but in various forms of mitochondrial dysfunction, such as defects in mitochondrial dynamics and transport, alterations in mitochondrial membrane permeability, dysregulated calcium homeostasis, mitophagy, and compromised ATP production. All these changes further amplify neurodegeneration and may trigger memory and learning impairment. Recent evidence from a mass spectrometry study found that pyruvate carboxylase, which catalyzes the conversion of pyruvate to oxaloacetate, was S-nitrosylated (and thus inhibited) in human postmortem brain samples from HIV-1-positive individuals with HAND and a history of METH use, but not in the HAND brains without METH abuse [46]. This data suggest that pyruvate metabolic processes and respiration may be affected to a greater extent in HAND in the setting of METH use. Moreover, another study demonstrated a reduction in the expression of TCA cycle-related genes, with an increase in glycolysis-related genes, in HIV-1 tg/METH self-administrating rats compared with wild type rats that self-administered METH [41]. The recently discovered association of OXPHOS pathway defects in peripheral blood mononuclear cells with neuroimaging measures of chronically HIV-1-infected individuals independent of METH use history suggested that this type of mitochondrial dysfunction may be more strongly linked to HIV-1 pathogenesis than to METH use [117].

Alterations in mitochondrial dynamics maintained by a balance of fission and fusion is believed to contribute to neurodegeneration. Indeed, any disbalance in mitochondrial dynamics may ultimately lead to the accumulation of either fragmented or hyperfused mitochondria. Dynamin-related protein 1 (DRP1) is the central molecular player that mediates mitochondrial fission. METH has been found to increase mitochondrial fragmentation through translocation of DRP1 in HIV-1-treated human primary neurons [50]. The impaired mitophagy detected in the presence of METH in combination with HIV-1 gp120 or Tat proteins in this study suggests its possible association with the accelerated aging observed in HIV-1-infected individuals. The fact that HIV-1 infection and METH dependence independently and adversely affected the leukocyte telomere to single copy gene ratio, a biomarker for aging and aging-associated diseases, supports this hypothesis [118]. Interestingly, DRP1 has been previously shown to be implicated in the pathogenesis of HAND [119] and HIV-1 encephalitis [120] in postmortem human brain studies, as well as in METH-mediated mitochondrial fragmentation in rat neural progenitor cells [121]. Recent research from our laboratory has presented evidence that exposure to METH increases HIV-1 infectivity in neural progenitor cells [122]. Additionally, our group reported that chronic exposure to METH resulted in enhanced proliferation of neural progenitor cells in the subventricular zone of the hippocampal dentate gyrus in an EcoHIV mouse model [37]. Continuous adult neurogenesis in the subgranular zone can play a neuroprotective role by replacing damaged neurons or glial cells with newly formed cells in response to such stimuli as BBB disruption [123]. METH-associated increase in HIV-1 infectivity of neural progenitor cells, in turn, might have negative long-lasting consequences for these cells, such as abnormal differentiation and spreading HIV-1 latency in the CNS.

Alterations in the dopaminergic system have long been associated with HIV-1 infection in the CNS [124]. METH, in turn, produces long-term damage to dopaminergic and serotonergic axon terminals in the striatum, hippocampus, and prefrontal cortex [42]. The dopamine D1 receptor (DRD1) and its targets were explored in METH-sensitized HIV-1 Tat tg rodent models. The nucleus accumbens, the brain region involved in reward processing and drug addiction, exhibited increased DRD1 immunoreactivity along with a significant increase in the addiction-associated transcription factor deltaFosB in METH self-administering HIV-1 tg rats, as compared with saline or METH-exposed wild-type rats [43]. In contrast, no changes in DRD1 stimulation or deltaFosB levels were found in the hippocampi of METH-treated HIV-1 tg rats [60], indicating region-specific alterations. The nucleus accumbens is crucial to drug-induced conditioned place preference and structural plasticity [125], and it has higher densities of DRD1-expressing neurons as compared to the hippocampus [126]. Furthermore, METH treatment markedly reduced dopamine D2 receptor (DRD2) and dopamine D5 receptor (DRD5) mRNA levels in the mouse striatum. This effect was further increased by Tat expression, especially in females exposed to METH [127]. These findings were consistent with the data from two recent meta-analyses that indicated significantly decreased D2/D3 receptor availability and transporter density in the striatum of METH users [128,129].

### 3.2. Contribution of Microglia and Astrocytes to Neuroinflammation

There is substantial evidence that inflammatory responses mediated by the activation of microglia are major drivers for the development and progression of HAND as well as METH-induced neurotoxicity [42,53,114]. A higher density of activated microglia within the temporoparietal cortex, a brain region important for numerous aspects of social cognition, was observed in HIV-1-infected individuals with lifetime METH dependence [22]. The findings from a recent gene expression profiling study showed a pronounced increase in the activation of immune- and inflammation-related pathways in the medial prefrontal cortex of HIV-1 tg rats with a history of escalated METH self-administration under long-access conditions [41]. One of the mechanisms that triggers microglia neurotoxic activity by METH in the context of HIV-1 could be related to a strong effect of METH on the transcription of genes associated with inflammation and chemotaxis pathways, especially CXCR4 and CCR5, which function as co-receptors for HIV-1 entry [44]. Moreover, METH potentiates HIV-1 gp120-induced microglia neurotoxic activity via voltage-gated potassium channel Kv1.3 and caspase 3/7 signaling [52]. Genes encoding functions in cell death pathways were shown to be overexpressed in microglia cells from the brains of rhesus monkeys with productive SIV infection treated with METH, as compared to SIV-infected animals not exposed to METH, and deficiencies in the brain-derived neurotrophic factor (BDNF) signaling pathway were predicted in this study [45]. In addition, the expression of the gene encoding suppressor cytokine signaling 1 (SOCS1), a marker of DNA damage and senescence, was significantly increased in SIV-infected microglia treated with METH as compared to SIV-infected microglia [44]. These METH-induced transcriptional changes confirm the role of METH abuse in promoting a proinflammatory environment in the brain.

Astrocytes provide trophic support for neurons and are involved in the regulation of transmission and synaptic activity, therefore an impairment in astrocyte function can negatively impact neurons. The mechanisms by which HIV-1 and METH lead to astrocytic neurotoxic activity are not entirely clear. A recent study demonstrated that acute treatment with HIV-1 led to the opening of mitochondrial permeability transition pores in the inner mitochondrial membrane in METH-cotreated primary human astrocytes [130]. Another study pointed to the induction of significant senescence of primary human fetal astrocytes mediated by the β-catenin pathway, which leads to neuronal toxicity in HIV-1 and METH comorbidity [38]. Emerging evidence suggests that cell senescence plays an important role in aging-associated diseases, including neurodegenerative diseases [131].

It has been proposed that METH use disorder exacerbates HAND partly through amplifying glutamate dysregulation of astrocytes, resulting in excessive release of glutamate to the CNS microenvironment. Chronic excess of extracellular glutamate acts as a neurotoxin, triggering neuronal oxidative stress and DNA damage [132]. When HIV-1-infected cultures of human neurons and astrocytes were treated with METH, a further increase in glutamate dysregulation, as well as increased apoptosis of both cell types was observed, and METH-induced changes in astrocytic gap junctions contributed to this process [49]. Recent work indicated that METH-induced downregulation of astrocytic excitatory amino acid transporter-2 (EAAT2), which is responsible for more than 90% of glutamate uptake from the synaptic environment, was exacerbated by interleukin-1β (IL-1β) in primary human astrocytes [133]. IL-1β has been shown to be important for HIV-1 pathobiology [102]. Moreover, EAAT2 was found to be S-nitrosylated in the HAND human brain [46]. The METH-induced EAAT2 dysfunction in astrocytes is mediated by trace amine associated receptor 1 (TAAR1). METH-induced transcription factor cyclic AMP response-binding protein (CREB) phosphorylation has been suggested as a critical mechanism for EAAT2 expression regulation, and may thus serve as a mechanistic target for the METH-induced TAAR1 activation, associated with impaired glutamate clearance capabilities in the context of HAND [133].

## 4. Combined Effects of HIV-1 Infection and Methamphetamine Use on Neurocognitive Functioning

Table 2 summarizes the recent human and rodent studies linking the combination of HIV-1 infection and METH use to the alteration of neurocognitive function and behavior. The human studies involved neuropsychological testing across various cognitive domains in adult participants with the presence or absence of HIV-1 infection and METH dependence. The results from a recent meta-analysis consisting of 37 studies suggested that working memory is perhaps the most significantly affected cognitive component of executive function among HIV-1-infected individuals in the ART era [134]. In animal studies, combined Tat expression and METH exposure increased perseverative errors during reversal learning in mice, suggesting impaired cognitive flexibility [135]. It was also indicated that administration of METH to HIV-1 Tat tg mice exacerbated the deficits in working memory and spatial learning characterized by decreased spontaneous alternations in the Y maze and increased latency time to reach the escape platform in Morris water maze tests, respectively [136]. Decreased levels of BDNF in all investigated brain regions could explain the impaired working memory seen in these experiments.

Sustained attention and vigilance are key components of situational awareness and underlie other cognitive functions, such as working memory and learning. While METH use has been found to be associated with sustained attention and vigilance deficits in humans, no interaction between HIV-1 and METH effects were found in a recent study [137]. Furthermore, prepulse inhibition (PPI) of the acoustic startle response, a measure of sensorimotor gating, has been reported to be the lowest in people with HIV-1 who also had a history of METH dependence [138]. These observations suggest that HIV-1 and METH may additively impair early information processing, potentially affecting downstream cognitive function. It has also been demonstrated that the lower levels of PPI were associated with worse performance on cognitive measures, tapping attention, and processing speed in patients with Parkinson’s disease [146].

Neurocognitive deficits are thought to substantially impact the ability to engage in the vital tasks of daily living. Although comorbid HIV-1 infection and METH dependence have been associated with worsening cognitive performance, additive effects of HIV-1 and METH were not observed for everyday functioning [139]. Results from two recent human studies demonstrated that individuals with comorbid HIV-1 and METH use disorder exhibited the highest rates of frailty (state of increased vulnerability to multisystem damage) and significantly poorer sleep quality compared to singly affected (HIV-/METH+ and HIV+/METH-) groups [140,141]. Moreover, disrupted sleep and frailty were associated with multiple adverse functional outcomes, including dependence in instrumental activities of daily living and poor ART adherence. Worse self-reported sleep was also found to be associated with greater hippocampal volume reduction across the adult lifespan [147]. In addition, sleep disturbances may be an underlying neurobiological mechanism of depression. While one study found the highest prevalence of lifetime major depression disorder in HIV+/METH+ individuals [142], the other study established that METH use was a more important determinant of depression than HIV-1 status [143]. In mouse studies, brain-specific Tat expression led not only to reward deficits, a core symptom of depression, but also to increasing METH-induced reward sensitivity [144,145]. However, it was also reported that the interaction between Tat and METH broadly prevented the METH-induced sensitization [148].

## 5. Developing New Therapeutic Approaches for Comorbid HIV-1 Infection and METH Use Disorder

With the great variety of impacts on the brain caused by HIV-1 infection, and further amplification when coupled with METH abuse, many novel treatments have arisen and continue to be developed to treat specific or multiple impairments. Depression was found to be decreased following consistent administration of mirtazapine [149], as well as by encouraging treatment adherence through the development of a personalized reminder text system [150]. The antioxidant N-acetylcysteine amide has been shown to diminish oxidative stress and protect against autophagy [36], while the steroid ginsenoside Rb1 has been observed to maintain BBB integrity in these circumstances [39]. Gastrodin, an active compound derived from an orchid, appears to be capable of protecting the cells of the NVU from a cascade of effects caused by HIV-1 infection and METH, including an increase in TJ protein expression to maintain BBB integrity [34]. The development of new nanoformulations is also creating unique treatment options, offering patients more targeted drug delivery options with lower risks of cytotoxic effects [88,151]. The simplest means of treatment may ultimately prove to be the most effective, as new programs to encourage adherence to existing treatment regimens through text messaging are proving to reduce detrimental behavior, as well as increase rates of adherence to treatment protocols [150,152,153,154].

## 6. Conclusions and Future Prospects

The combined effects of HIV-1 infection and METH use on the NVU can be deleterious and contribute to the clinical complexity of HAND. Indeed, HIV-1 infection and METH use disorder have a striking synergistic impact on NVU dysfunction and BBB damage, by inducing vascular oxidative stress, increasing the degradation of TJs and basement membrane proteins, and inhibiting nutrient and drug transporters. The impairments in astrocytic glutamate clearance and in the release of proinflammatory mediators and neurotrophic factors are potentiated in HIV-1 and METH comorbidity, and may lead to compromised synaptic functions along with neuronal cell death. The synergistic induction of mitochondrial dysfunction and apoptosis by HIV-1 and METH occurs in both neurons and astrocytes, despite the fact that these NVU cell types preferentially use different metabolic pathways. Disruption of the BBB by the combination of METH and HIV-1 may be related to the release of proinflammatory and neurotoxic mediators not only by glial cells but also by pericytes. Thus, future studies examining the mechanisms of BBB disruption and neuroinflammation mediated by pericytes during co-morbid METH abuse and HIV-1 infection are warranted.

A focus on the NVU for potential drug targets may be helpful to develop novel NVU-targeted therapies in HAND aggravated by METH use. In this regard, TRPM2 channels in brain endothelial cells may serve as a promising drug intervention target to reduce BBB injury and neuropsychiatric symptoms in HIV-1-infected METH abusers. Targeting the EAAT2/TAAR1 pathway in astrocytes could also represent a promising strategy to reduce the accumulation of extracellular glutamate and ameliorate neuronal injury. There is a strong need for in vivo studies addressing the association not only between cognitive impairment and neuronal injury induced by the combination of METH use and HIV-1 but also between BBB breakdown and cognitive disturbances in this pathology. Although most of the studies analyzed in this review were performed in the absence of ART, it is imperative for future studies to characterize the interactive effects of not only METH and HIV-1, but also of antiretroviral therapeutics.

The coupling of METH misuse with HIV-1 infection is characterized by worsening neurocognitive deficits in executive functions, working memory, and to a lesser degree in learning. Impairment in executive functions such as planning or self-regulation may contribute to medication nonadherence, whereas the association between METH use and risky sexual behavior can be mediated by impaired executive functions in HIV-1 infection. Moreover, alterations in dopamine receptor density or availability that are synergistically induced by METH use and HIV-1 may be especially harmful because they can promote addictive behaviors, such as impulsivity or cravings, and facilitate the development of depression symptoms. The findings from these studies lay the groundwork for future longitudinal research, examining whether frailty and disrupted sleep predict onset of neurocognitive decline in individuals with comorbid HIV-1 and METH use disorder. Furthermore, psychosocial treatments as well as the use of a variety of emerging technologies, such as online social networking and mobile technologies, should be utilized in conjunction with pharmacotherapy to target METH use and improve cognitive functioning in HAND.

## Figures and Tables

**Table 1:** A summary of recent studies showing that HIV-1 infection and methamphetamine (METH) use synergistically impair the neurovascular unit (NVU).

Study and Year	Experimental Model	Viral Inoculum Dose/Route	METH Dosing Regimen	Synergistic Effects on NVU
Combined In Vivo and In Vitro Studies				
Huang et al., 2021 [34]	Tree shrews; HCMEC/D3 cell line (human)	Tat (100 ng) by tail i.v. injection; 25 to 200 nM of Tat	8 mg/kg i.p. for 10 consecutive days; 0.05 to 2.0 mM for 24 h	Enhanced BBB permeability due to alterations in TRPM2 channels and TJ protein expression both in vivo and in vitro
Li et al., 2021 [35]	Tree shrews; HCMEC/D3 cell line (human)	Tat (100 ng) by tail i.v. injection; 100 nM of Tat	8 mg/kg i.p. for 10 consecutive days; 500 μM for 24 h	Decreased expression of TJ proteins and increased BBB permeability both in vivo and in vitro; Downregulation of GLUT1 and GLUT3 protein expression both in vivo and in vitro
Zeng et al., 2018 [36]	Rats; SH-SY5Y neuroblastoma cell line (human)	Tat (50 ng/kg) by tail i.v. injection; 50 and 100 nM of Tat	10 mg/kg i.p. for 7 consecutive days; 1 and 2 mM for 24 h	Exacerbation of oxidative stress both in vivo and in vitro
Park et al., 2021 [37]	C57BL/6 mice; human primary neural progenitor cells	EcoHIV (1 μg of p24) via left internal carotid artery injection; HIV-1 NL4-3 (60 ng/mL of p24)	Escalating dose regimen for 6 days: 1.0–4.0 mg/kg i.p.; 100 μM for 24 h	Enhanced neural progenitor cell proliferation both in vivo and in vitro
Yu et al., 2017 [38]	HIV-1 transgenic rats; primary human fetal astrocytes	N/A; HIV-1 BaL (10 ng or 20 ng of p24)	10 mg/kg i.p. every 2 h for 4 days; 10, 30, 100, 300 and 1000 μM daily for 5 days	Induction of astrocyte senescence both in vivo and in vitro
**In Vivo Studies**				
Li et al., 2018 [39]	Sprague-Dawley rats	Tat (50 ng) i.c.v.	10 mg/kg i.p. for 7 consecutive days	Decreased expression of TJ proteins and increased BBB permeability; Exacerbation of oxidative stress and neuronal damage
Hoefer et al., 2015 [40]	HIV-1 gp120 transgenic mice	N/A	Escalating dose multiple-binge regimen for 25 days: 0.1–6.0 mg/kg s.c.	Reduction in post-tetanic potentiation in hippocampal slices; Decreased dendritic spine density
de Guglielmo et al., 2015 [41]	HIV-1 transgenic rats	N/A	Escalating dose multiple-binge regimen for 15 consecutive sessions: 0.5 mg/kg/0.1 mL 6 h/day i.v.	Gene expression changes indicative of an increase in neuronal damage and impaired aerobic glucose metabolism in the medial prefrontal cortex
Ohene-Nyako et al., 2018 [42]	HIV-1 transgenic rats	N/A	0.02–0.04 mg/kg/0.05 mL i.v. infusion 2 h/day for 21 days	Upregulation of DRD1 and deltaFosB expression in the nucleus accumbens
Baek et al., 2020 [43]	Doxycycline-inducible HIV-1 Tat transgenic mice	N/A	2 mg/kg i.p. once a day for 7 days (acquisition phase); 1 mg/kg (challenge phase)	Reduction in DRD2 and DRD5 mRNA levels in the striatum
Najera et al., 2016 [44]	Rhesus macaques	SIVmac251 i.v. (infectious dose was not reported)	Escalating dose regimen for 23 weeks with a final dose of 2.5 mg/kg i.m.	Upregulation of genes encoding proteins involved in DNA damage and senescence in microglia
Niu et al., 2020 [45]	Rhesus macaques	SIVmac251 i.v. (infectious dose was not reported)	Escalating dose regimen over a month-long period: 0.1–2.5 mg/kg i.m.	Upregulation of genes encoding proteins involved in cell death pathways and deficiencies in the BDNF-signaling pathway in brain microglia/macrophages
**Postmortem Human Brain Ex Vivo Studies**				
Soontornniyomkij et al., 2016 [22]	Human postmortem brain samples	N/A	Lifetime METH dependence	Focal cerebral microgliosis
Doulias et al., 2021 [46]	Human postmortem brain samples	N/A	Duration of METH use was not reported	Increase in S-nitrosylation of tricarboxylic acid enzymes
**In Vitro Studies**				
Patel et al., 2017 [47]	HCMEC/D3 cell line (human)	Tat (100 nM)	10 μM for 24 h	Reduced ZO-1 TJ protein expression (in line with in vivo studies [34,35,39]); Increased rhodamine 123 accumulation
Cao et al., 2016 [48]	Simian virus 40 (SV40)-transformed astrocyte cell line (human)	gp120 (400 pM)	500 μM for 24 h	Autophagy initiation
Castellano et al., 2016 [49]	Human primary mixed cultures of neurons and astrocytes	HIV-1 ADA (infectious dose was not reported)	1 and 10 μM for 7,14 and 21 days	Enhancement of apoptosis
Teodorof-Diedrich et al., 2020 [50]	Human primary neurons	gp120, Tat or gp120/Tat (100 ng/mL)	300 μM for 24 h	DRP1-dependent mitochondrial fragmentation; Neurite length reduction (in line with in vivo study [50])
Li et al., 2018 [51]	Tree shrew primary midbrain neuronal cells	Tat (50 nM and 100 nM)	0.1–0.5 mM at varying time periods	Autophagy initiation
Liu et al., 2017 [52]	Cultured rat microglial cells	gp120 (0.1, 0.5 and 1.5 nM)	2, 20, and 200 µM for 24 h	Induced KV1.3 potassium channel- mediated microglial neurotoxicity; Increased caspase-3/7 activity in microglia (in line with in vivo studies [52,53])

BBB: blood–brain barrier; BDNF: brain derived neurotrophic factor; DRD1: dopamine receptor D1; DRD2: dopamine receptor D2; DRD5: dopamine receptor D5; DRP1: dynamin-related protein 2; FOXO3: forkhead box O transcriptional factor; GLUT1: glucose transporter 1; GLUT3: glucose transporter 3; i.c.v.: intracerebroventricular; i.m.: intramuscular; i.p.: intraperitoneal; i.v.: intravenous; METH: methamphetamine; NVU: neurovascular unit; ROS: reactive oxygen species; s.c.: subcutaneous; TJ: tight junction; TRPM2: transient receptor potential melastatin 2; ZO-1: zona occludens-1.

**Table 2:** A summary of recent studies exploring the alterations in neurocognitive functioning and behavior due to the combination of HIV-1 infection and methamphetamine (METH) use.

Study and Year	Study Design	Experimental Model	Study Outcomes
Human studies			
Pocuca et al., 2020 [137]	Cross-sectional	205 adults (67 HIV-/METH-, 36 HIV-/METH+, 49 HIV+/METH-, and 53 HIV+/METH+)	METH, but not HIV-1, was associated with sustained attention and vigilance deficits.
Walter et al., 2021 [138]	Cross-sectional	205 adults (69 HIV-/METH-, 40 HIV-/METH+, 52 HIV+/METH-, and 44 HIV+/METH+)	Prepulse inhibition was most decreased in people with HIV-1 and a history of METH dependence.
Minassian et al., 2017 [139]	Cross-sectional	172 adults (49 HIV-/METH-, 48 HIV-/METH+, 37 HIV+/METH-, and 38 HIV+/METH+)	Additive effects of HIV-1 and METH were not observed for everyday functioning.
Paolillo et al., 2019 [140]	Cross-sectional	210 adults (92 HIV-/METH-, 75 HIV+/METH-, and 43 HIV+/METH+)	Persons with comorbid HIV-1 and METH use disorder had higher frailty index scores than both HIV-/MA- and HIV+/MA- participants. Additional models linked higher frailty index scores to worse global neurocognition and greater likelihood of everyday functioning dependence among the HIV+/METH+ group.
Sun-Suslow et al., 2020 [141]	Cross-sectional	313 adults (72 HIV-/METH-, 16 HIV-/METH+, 141 HIV+/METH-, and 84 HIV+/METH+)	HIV+/METH+ individuals endorsed significantly more problematic sleep than HIV+ and HIV-/METH- individuals. Poorer reported sleep quality among HIV+/METH+ was also associated with multiple adverse functional outcomes including greater objective cognitive impairment.
Saloner et al., 2020 [142]	Cross-sectional	125 adults (23 HIV-/METH-, 35 HIV-/METH+, 22 HIV+/METH-, and 45 HIV+/METH+)	Prevalence of lifetime major depression disorder was higher in HIV+/METH+ compared with the other groups.
Javanbakht et al., 2020 [143]	Longitudinal	534 men (267 HIV+ and 267 HIV-); METH use was not individually reported	Frequent METH use, but not HIV status, was associated with persistence of depressive symptoms.
**Rodent Studies**			
Kesby et al., 2018 [135]	Cross-sectional	Doxycycline-inducible HIV-1 Tat transgenic mouse model	The combination of Tat expression and METH exposure did not induce significant learning deficits but did increase perseverations at the initiation of reversal learning suggesting slight impairments in executive function.
Nookala et al., 2018 [136]	Cross-sectional	Doxycycline-inducible HIV-1 Tat transgenic mouse model	Administration of METH to HIV-1 Tat transgenic mice exacerbated the deficits in spatial learning and memory characterized by decreased spontaneous alternations in Y maze and increased latency time to reach the escape platform in the Morris water maze.
Walter et al., 2021 [138]	Cross-sectional	Doxycycline-inducible HIV-1 Tat transgenic mouse model	Chronic METH treatment and Tat expression did not interact to affect prepulse inhibition in mice.
Kesby et al., 2016 [144]	Cross-sectional	Doxycycline-inducible HIV-1 Tat transgenic mouse model	Tat expression in mice led to reward deficits, a core symptom of depression, and a greater sensitivity to METH-induced reward enhancement.
Kesby et al., 2019 [145]	Cross-sectional	Doxycycline-inducible HIV-1 Tat transgenic mouse model	Longer-term Tat expression, or its induction at earlier stages of METH exposure, was more consequential at inducing behavioral and neurochemical effects.

## Data Availability

Not applicable.
